# Bursal Enlargement of the Tendon of the Flexor Pedis Perforans

**Published:** 1883-07

**Authors:** 


### III.—BURSAL ENLARGEMENT OF THE TENDON OF THE FLEXOR PEDIS PERFORANS.

REPORTED FROM DR. BERNS CLINIC HELD AT HIS PRIVATE HOSPITAL.

The subject in hand was a young horse aet six, with no previous history, except that the animal had had an enlarged hock for an indefinite period of time.

The greatly enlarged tarsus was carefully examined by the senor students, and they advanced the opinion that the fluid should be withdrawn. Dr. Berns was also of the same opinion, and made an incision into the tumor, and one-half pint of fluid escaped. The cavity was then injected with a solution of carbolic acid.

The Doctor did not apprehend any serious consequences, and therefore gave a favorable prognosis.

For ten days after the operation the serum continued to escape. During the first five days about four quarts were discharged daily. The hock diminished in size, but there was not the marked improvement looked for.

After this the hock was blistered, without decided result.

Although the operation and after treatment did not accomplish all that could be desired, there was some improvement after a long time.

